# Intracellular trafficking of hyaluronic acid-chitosan oligomer-based nanoparticles in cultured human ocular surface cells

**Published:** 2011-01-27

**Authors:** Laura Contreras-Ruiz, María de la Fuente, Jenny E. Párraga, Antonio López-García, Itziar Fernández, Begoña Seijo, Alejandro Sánchez, Margarita Calonge, Yolanda Diebold

**Affiliations:** 1 Ocular Surface Group-IOBA, University of Valladolid, Valladolid, Spain; 2 Networking Research Center on Bioengineering, Biomaterials and Nanomedicine (CIBER BBN), Valladolid, Spain; 3 NANOBIOFAR Group, Department of Pharmaceutical Technology, University of Santiago de Compostela, Santiago de Compostela, Spain

## Abstract

**Purpose:**

Nanoparticles are a promising alternative for ocular drug delivery, and our group has proposed that they are especially suited for ocular mucosal disorders. The goal of the present study was to determine which internalization pathway is used by cornea-derived and conjunctiva-derived cell lines to take up hyaluronic acid (HA)-chitosan oligomer (CSO)-based nanoparticles (HA-CSO NPs). We also determined if plasmids loaded onto the NPs reached the cell nucleus.

**Methods:**

HA-CSO NPs were made of fluoresceinamine labeled HA and CSO by ionotropic gelation and were conjugated with a model plasmid DNA for secreted alkaline phosphatase. Human epithelial cell lines derived from the conjunctiva and the cornea were exposed to HA-CSO NPs for 1 h and the uptake was investigated in living cells by fluorescence microscopy. The influence of temperature and metabolic inhibition, the effect of blocking hyaluronan receptors, and the inhibition of main endocytic pathways were studied by fluorometry. Additionally, the metabolic pathways implicated in the degradation of HA-CSO NPs were evaluated by lysosome identification.

**Results:**

There was intracellular localization of plasmid-loaded HACSO NPs in both corneal and conjunctival cells. The intracellular presence of NPs diminished with time. HA-CSO NP uptake was significantly reduced by inhibition of active transport at 4 °C and by sodium azide. Uptake was also inhibited by blocking hyaluronan receptors with anti-CD44 Hermes-1 antibody, by excess HA, and by filipin, an inhibitor of caveolin-dependent endocytosis. HA-CSO NPs had no effect on cell viability. The transfection efficiency of the model plasmid was significantly higher in NP treated cells than in controls.

**Conclusions:**

HA-CSO NPs were internalized by two different ocular surface cell lines by an active transport mechanism. The uptake was mediated by hyaluronan receptors through a caveolin-dependent endocytic pathway, yielding remarkable transfection efficiency. Most of HA-CSO NPs were metabolized within 48 h. This uptake did not compromise cell viability. These findings further support the potential use of HA-CSO NPs to deliver genetic material to the ocular surface.

## Introduction

Gene therapy can be broadly defined as the introduction of genetic material into a cell for either the suppression of gene expression or the production of a needed protein. Because the eye has well defined anatomy, immune privilege, and accessibility, it is a promising candidate organ to benefit from gene therapy. The ocular surface is covered by two protective mucosal epithelia, the cornea and conjunctiva. These epithelia are in direct contact with the tear film and act as barriers for topically administered drugs. Along with physiologic mechanisms such as blinking and tear clearance, the epithelia limit the efficient penetration of drugs and DNA into the eye.

To achieve efficient delivery of DNA to mucosal cell nuclei, several barriers must be overcome. Among the different strategies explored to improve mucosal delivery, one of the most promising is the use of mucoadhesive nanoparticles (NPs) that are capable of interacting with the ocular mucosa. This interaction increases drug residence time and promotes its transport across the ocular barriers [1]. Our group has developed NPs consisting of bioadhesive and biocompatible polysaccharides, such as chitosan (CS) and hyaluronic acid (HA), intended for gene delivery to the ocular surface [2,3]. CS is a non-toxic and biocompatible cationic polysaccharide with several applications for the administration of drugs and genes [4]. CS NPs interact and remain associated with the ocular mucosa for extended periods of time [5], increasing the delivery to external ocular tissues, and providing long-term drug retention [6]. In contrast, HA is an acidic mucopolysaccharide distributed widely in the eye. It has been used for the preparation of microparticles [7] and as a coating material for preformed liposomes [8], NPs [9], and plasmid DNA (pDNA) complexes [10]. In previous studies by our group [2], we have shown that NPs of HA and oligomers of CS (HA-CSO NPs) have the ability to associate with significant amounts of plasmid pDNA, enter cells, and efficiently deliver the pDNA. In rabbits, these NPs entered conjunctival and corneal epithelial cells without causing ocular discomfort or irritation and without significant effects on tissue morphology and functionality or tear production or drainage [11].

Improving gene delivery requires developing an understanding of the cellular uptake mechanisms, intracellular stability, and bioavailability of the therapeutic DNA. Five major cell uptake mechanism are distinguished [12]: a) macropinocytosis; b) phagocytosis; c) clathrin-dependent endocytosis; d) caveolin-mediated endocytosis; and e) clathrin- and caveolin-independent pathways. Macropinocytosis and phagocytosis are actin-dependent endocytic mechanisms mainly used by cells to internalize large amounts of fluids and growth factors (macropinocytosis) or solid particles (phagocytosis). Endocytosis mediated by clathrin and caveolins comprises multiple mechanisms that allow cells to internalize macromolecules and particles into transport vesicles derived from the plasma membrane [13]. Clathrin-dependent endocytosis enables cargo bound to specific membrane-bound receptors to be internalized [14]. Caveolins are the main protein component of caveolae, flask-shaped invaginations of the plasma membrane that participate in macromolecule internalization [15]. Finally, clathrin- and caveolin-independent pathways transport cargo to the glycosylphosphatidylinositol-anchored-protein-enriched early endosomal compartment [16].

The presence of HA in the HA-CSO NPs may facilitate NP cellular uptake by receptor-mediated endocytosis. HA is biocompatible, biodegradable, and mucoadhesive, and affects several cellular processes. For instance, HA promotes corneal wound healing through regulation of epithelial cell regeneration and migration. This regulation is achieved by binding of HA to two receptors, CD44 and the receptor for hyaluronan-mediated motility (RHAMM) located in ocular surface epithelia [17]. These receptor-mediated processes may be implicated in the cellular uptake of HA-CSO NPs.

In addition to internalization mechanisms, the intracellular trafficking of DNA-loaded NPs and bioavailability of the transported DNA within the cell are critical elements to study when a new drug carrier is proposed. Among these issues, the degradation of the NPs in the lysosomes following their internalization is a key point [18]. Lysosomes are the terminal degradative compartment of certain endocytic pathways [19] and may negate effective drug targeting, in this case of pDNA, to the nucleus.

We propose that HA-CSO NPs may serve as an effective gene delivery system for ocular surface disorders. Therefore, the goal of the present work was to study the intracellular trafficking of HA-CSO NPs loaded with a model pDNA. We aimed to determine which HA-CSO NP internalization pathways are used by corneal and conjunctival cells and whether or not pDNA reaches the cell nucleus.

## Methods

### Materials

Plastic culture ware was obtained from Nunc (Roskilde, Denmark). DMEM/F12 culture medium and other cell culture reagents were from Invitrogen-Gibco (Inchinnan, UK). Reagents 2,3-bis[2-methoxy-4-nitro-5sulfophenyl]-2H-tetrazolium-5-carboxyanilide inner salt (XTT), sodium azide, colchicine, chlorpromazine, and filipin were purchased from Sigma-Aldrich Corp. (St. Louis, MO). For immunfluorescence studies, we used anticaveolin-1 (BD Bioscience, San Jose, CA) and anti-clathrin (Affinity Bioreagents, Rockford, IL) primary monoclonal antibodies (Abs) and goat anti-mouse IgG F(ab´)2 PE-Cy5 (Santa Cruz Biotechnology, Santa Cruz, CA) as a secondary antibody. The Vibrant™Phagocytosis assay kit and LysoSensor® reagent were obtained from Molecular Probes (Leiden, The Netherlands). The fluorimetric Sensolyte® FDP Secreted Alkaline Phosphatase Reporter Gene Assay was purchased from Anaspec (Fremont, CA) and the kit Label IT IT® Cy™3 from Mirus Bio LLC (Madison, WI).

### Preparation of HA-CSO NPs

HA-CSO NPs were made of the polysaccharides HA and CSOs by a slightly modified ionotropic gelation technique [2]. Briefly, 375 µl of HA solution (0.73 mg/ml), mixed with 50 µl of the crosslinker tripolyphosphate (TPP, 0.59 mg/ml), was added over 750 µl of CSO solution (0.625 mg/ml) with magnetic stirring. HA was previously labeled with fluoresceinamine (fl-HA). CSOs were obtained by sodium nitrite degradation as previously described by Janes et al. [20]. Briefly, 200 μl of 0.1 M NaNO_2_ were added to 4 ml of a 10 mg/ml chitosan solution. The reaction was left overnight to complete the degradation, and oligomers of approximately 10–12 kDa were recovered by freeze-drying. HA-CSO NPs were loaded with a model pDNA encoding secreted alkaline phosphatase (SEAP) by incorporating the pDNA in the HA/TPP. The theoretical loading was set at 1% (W/W). The SEAP plasmid was labeled with cyanine-3 (Cy-3), using the kit Label IT IT® Cy™3.

### Physicochemical characterization and pDNA loading of HA-CSO NPs

Mean particle size and size distribution (polidispersity) were measured by photon correlation spectroscopy. Also, Z-potential values were obtained by laser Doppler anemometry. Stability of NPs upon storage at 4 °C for up to two weeks was evaluated by periodically measuring the size and Z-potential.

### Cell lines and culture conditions

Three different cell lines were used. The IOBA-NHC cell line is a nontransfected, spontaneously immortalized conjunctival epithelial cell line [21] used at passages 71 to 87. Cells were grown in DMEM/F-12 supplemented with 10% fetal bovine serum (FBS), 5,000 U/ml penicillin, 5 mg/ml streptomycin, 2.5 μg/ml fungizone, 2 ng/ml human epidermal growth factor (EGF), 1 µg/ml bovine insulin, 0.1 µg/ml cholera toxin, and 0.5 µg/ml hydrocortisone.

The HCE cell line is a SV40-immortalized human corneal epithelial cell line [22]. Cells from passages 42 to 52 were cultured in DMEM/F-12 supplemented with 15% FBS, 100 U/ml penicillin, 0.1 mg/ml streptomycin, 10 ng/ml EGF, 0.5% DMSO, 5 μg/ml insulin, and 0.1 µg/ml cholera toxin.

The mouse macrophage RAW264.7 cell line [23] was cultured in RPMI 1640 supplemented with 10% FBS, 5% L-glutamine, 5% penicillin, and 5% streptomycin. It was used as a control in the phagocytosis assay.

All cell lines were cultured at 37 °C in a 5% CO_2_%–95% air atmosphere. Media were changed every other day, and daily observations were made by phase contrast microscopy.

### Cell viability assay

Viability of 20 µg/ml HA-CSO NP-exposed cells was measured using the XTT toxicity test [24]. Cells were seeded onto 96-well plates (2×10^5^ cells/well) and grown until 75% confluence. Culture medium was replaced with fresh phenol red-free RPMI, 48 h after NP incubation. Then XTT solution was added and cells incubated at 37 °C for 15 h. Plates were read in a SpectraMAX®M5 multidetection microplate reader (Molecular Devices, Sunnyvale, CA) at 450 nm (reference wavelength: 620 nm). Controls included cells alone and cells exposed to 0.5% benzalkonium chloride (BKC), which induces a significant decrease of ocular cell viability in concentrations greater that 0.05% [25]. Cell viability was calculated as a percentage with regard to control cells. Each test was repeated four times in triplicate.

### Uptake and trafficking experiments

Corneal and conjunctival cell lines were seeded in eight-well multichamber Permanox™ slides (5×10^5^ cells/well) and 24-multiwell plates (8×10^5^ cells/well). When the confluent state was reached, cells were washed out in supplement-free culture medium for 1 h, and 100 µl of 20 µg/ml HA-CSO NPs were then added. After 1 h incubation, the cells were washed three times with phosphate buffered saline (PBS) with 0.27% glucose, and fresh medium was added. HA-CSO NP uptake was monitored after 1, 6, 24, and 48 h by fluorescence microscopy and by fluorometry. Each assay was performed four times in triplicate.

For fluorescence microscopy, living cells were viewed under an inverted fluorescence microscope (Leica DMI 6000B, Wetzlar, Germany). Cell nuclei were counterstained by Hoescht dye. To assure the intracellular location of the NPs, vertical spatial images were generated by Z-scans in 1 µm steps for 20 images. For fluorometry, the cells were washed to remove any extracellular NPs, then frozen, thawed, and disrupted with 100 µl ice-cold radioimmunoprecipitation assay (RIPA) buffer (10 mM Tris-HCl [pH 7.4], 150 mM NaCl, 1% deoxycholic acid, 1% Triton X-100, 0.1% SDS, and 1 mM EDTA). Fluorescence in cell lysates was measured in a SpectraMAX®M5 multidetection microplate reader at an excitation wavelength of 490 nm and an emission wavelength of 520 nm. The amount of NPs taken up by the cells was expressed in arbitrary units of fluorescence intensity. Negative controls included cell incubation with Hank's balanced salt solution instead of NPs.

To explore different mechanisms of NP interaction with epithelial cells and trafficking across the plasma membrane, uptake of HA-CSO NPs was studied under seven different blocking conditions (Table 1). In all cases, cells were incubated with the different inhibitors for 30 min before the addition of the NPs, and then co-incubated with the NPs for 1 h.

**Table 1:** Uptake pathways and blocking conditions.

**Pathway**	**Inhibitor**	**Mechanism**	**References**
Phagocytosis	-	Vibrant™Phagocytosis assay kit (fluorescent *E. coli* bioparticles)	[43]
Energy dependent processes	4 °C	Blocks energy dependent processes	[44]
	Sodium azide (100 mM)	Inhibits ATP production.	[45]
	Colchicine (15 mM)	Inhibits endocytic processes	[46]
HA-receptor-mediated pathway	Hermes-1 (1 µg/100 µl)	Blocks HA receptor CD-44	[47]
	Excess of HA (50×)*	Blocks HA receptors	[47]
Endocytosis	Filipin (1.25 µg/ml)	Specifically inhibits caveolin-mediated endocitosis	[48]
	Chlorpromazine (25 µM)	Specifically inhibits clathrin-mediated endocytosis	[49]

*50 fold with respect to the amount of HA forming the NPs.

The phagocytic capacity of both the corneal and conjunctival cell lines was studied to exclude phagocytosis as a HA-CSO NP uptake pathway. The phagocytosis assay was performed using the Vibrant™Phagocytosis assay kit following the manufacturer’s protocol. RAW264.7 cells were used as control. Briefly, HCE, IOBA-NHC, and RAW264.7 cells were grown in 96-well plates until confluence. Fluorescent *Escherichia coli* bioparticles were opsonized by incubation with human serum for 1 h at 37 °C. The fluorescent bioparticle suspension was added to the cultures and incubated at 37 °C. Negative controls included incubation without bioparticles. After 2 h, the bioparticle suspension was removed and a Trypan blue solution (0.25 mg/ml) was used to quench the extracellular fluorescence. The fluorescence associated with the phagocytosed bioparticles inside the cells was measured in a SpectraMAX®M5 multidetection microplate reader at an excitation wavelength 480 nm and an emission wavelength of 529 nm. Results were expressed as relative fluorescence units (RFU). Each assay was performed five times in triplicate.

### Immunofluorescence assays

HCE and IOBA-NHC cells were seeded onto eight-well multichamber Permanox™ slides (5×10^5^ cells/well), grown until confluence, and incubated with 100 µl of 20 µg/ml HA-CSO NPs. After 1 h, the cells were washed three times with 0.27% glucose in PBS and fixed in ice-cold methanol. After several washes with PBS, they were incubated at room temperature (RT) for 50 min with blocking buffer composed of PBS with 4% goat serum, 0.3% Triton X-100, and 1% BSA to block the non-specific binding. Then they were incubated with anti-caveolin-1 (0.25 µg/ml) or anti-clathrin antibody (10 µg/ml) at 37 °C for 1 h. After several washes with PBS, cells were incubated with PE-Cy5-conjugated secondary antibodies (4 µg/ml) for 1 h at RT. Cell nuclei were counterstained by Hoescht dye. The preparations were viewed under an epifluorescence microscope. Each experiment was performed three times, and negative controls included the omission of primary antibodies.

### Degradative pathways

Lysosomes in living cells were labeled and tracked by the fluorescent acidotrophic probe LysoSensor®. HCE and IOBA-NHC cells were grown on eight-well multichamber Permanox™ slides (5×10^5^ cells/well) until confluence and then incubated with HA-CSO NPs for 1 h. After incubation, the medium was removed and Lysosensor® (150 nM in DMEM culture medium) was added. Cells were incubated for 3 min in 5% CO_2_ at 37 °C. Before observation by microscopy, the solution was replaced with fresh DMEM medium.

### Transfection assays

The yield of gene expression was evaluated by monitoring concentrations of SEAP in cells exposed to pSEAP-loaded HA-CSO NPs, using the fluorimetric Sensolyte® 3,6-fluorescein diphosphate (FDP) SEAP reporter gene assay. Samples of culture medium were prepared according to the manufacturer protocols 48 h after NP incubation. The fluorogenic phosphatase substrate FDP was used to assess the activity of generic phosphatase activity. Fluorescein, the final hydrolytic product, was quantified by excitation at 485 nm and emission at 528 in a SpectraMAx®M5 multidetection microplate reader. Each assay was performed four times in triplicate.

### Statistical analysis

Statistical analyses were performed by a biostatistician (co-author I.F.). Results were expressed as means±standard error of the means. The statistical significance between control NP treatment and inhibitor effects at each time point was analyzed by ANOVA with three fixed effects, using the Box-Cox transformation with λ=-1 to verify the assumptions of normality, variance homogeneity, and independence. Data from phagocytosis, transfection, and viability assays were analyzed by ANOVA with two fixed effects. After performing a Levene's test to assess the equality of variances and a variance decomposition to determine how much of the forecast error variance could be explained by the model, pairwise comparisons were performed. Differences were considered to be significant when p≤0.05.

## Results

### Nanoparticle characterization

The complete physico-chemical and morphological characterization of this kind of NP has been reported [26]. The mean size of the HA-CSO NPs was 104.6±0.2 nm, with a positive Z-potential of 31±0.6 mV, and a polydispersity index of 0.167±0.022. Neither particle size nor the Z-potential changed when the NPs were stored at 4 °C for up to 2 weeks (data not shown). Thus the NPs had good stability during storage.

### Uptake of plasmid-loaded HA-CSO NPs

**Figure 1:**
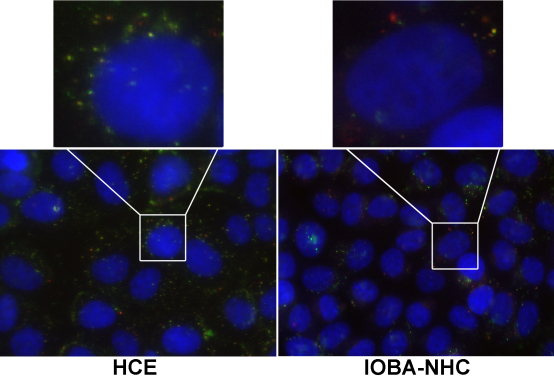
Fluorescence micrographs of HA-CSO NP uptake (6 h). Fluoresceinamine-HA (green) and Cy3-plasmid (red) were evident in the cytoplasm of HCE and IOBA-NHC cells. Nuclei were counterstained with Hoescht dye (blue). Insets show higher magnification of cytoplasmic NPs. Representative images of four different experiments are shown. (Magnification 63×, inset magnification 252×).

Uptake of HA-CSO NPs in the cultured corneal and conjunctival cell lines was studied by fluorescence microscopy. After HA-CSO NP incubation, the NPs appeared as nano-sized fluorescent dots inside the cells (Figure 1). The green fluorescence was attributed to the fl-HA and the red fluorescence was attributed to the Cy3-plasmid. Control cells exposed to Hank’s balanced salt solution showed neither green nor red fluorescence, as expected. In general, cells incubated with HA-CSO NPs had a well preserved morphology that was similar to controls. There was a sustained intracellular localization of plasmid-loaded HA-CSO NPs in both corneal and conjunctival cell lines (Figure 1). Uptake was always higher in corneal cells compared to the conjunctival cells. Plasmid associated fluorescence (red) was localized in the nucleus or in the perinuclear region after 24 and 48 h in both cell lines. At these times, most of the red fluorescence was independently identified from the green fluorescence. Therefore, it can be assumed the plasmid separated from the NPs and reached the nucleus. Red-dotted fluorescence was more easily identified in HCE than in IOBA-NHC cells. Different Z-stacks confirmed the intracellular localization of plasmid and NPs.

**Figure 2:**
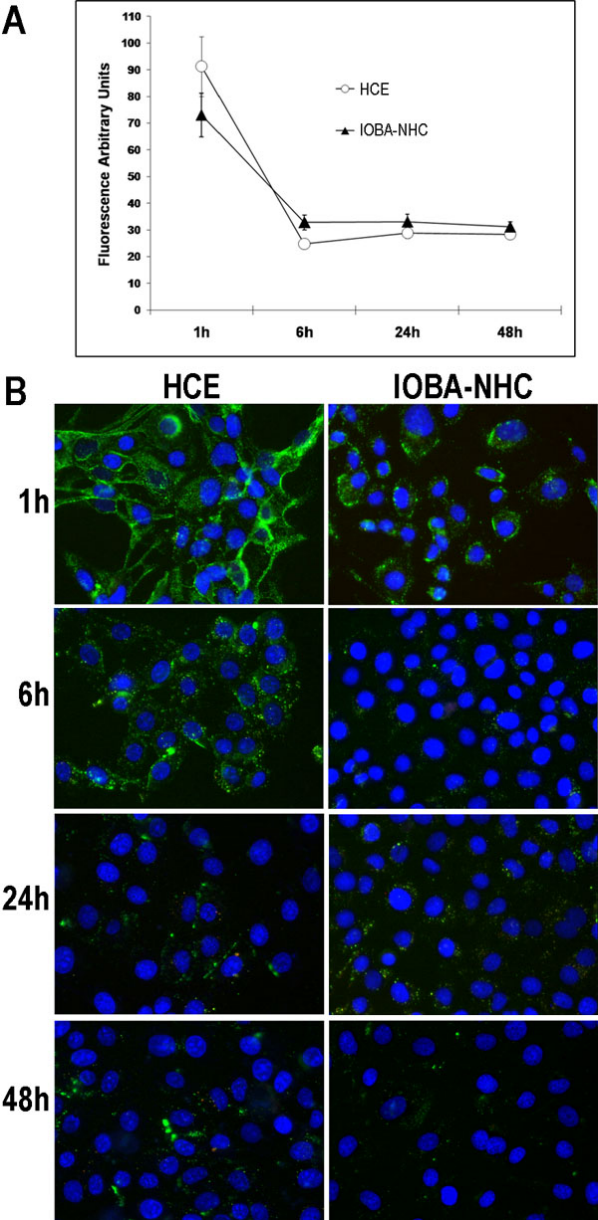
Persistence of HA-CSO NPs over time. **A**: For both corneal and conjunctival cells, fluorescence intensity was greatest at 1 h after incubation. By 6 h, the intensity diminished to approximately half, where it remained stable for up to 48 h. **B**: NP-associated fluorescence diminished with time, as fluorescence micrographs at 1, 6, 24, and 48 h after treatment show. Uptake was higher in the HCE than in the IOBA-NHC cells (n=4).

The fluoresceinamine fluorescence signal became reduced and more dispersed as the time of incubation increased (Figure 2), suggesting a process of intracellular degradation. Cellular retention of NP-associated fluorescence was quantified by fluorometry at 1, 6, 24, and 48 h. After 1 h, uptake was confirmed in both cell lines, showing corneal cells with higher cytoplasmic fluorescence levels than conjunctival cells (Figure 2), although the differences were not significant. The intracellular presence of NPs diminished with time, with significantly higher levels at 1 h compared to 6, 24, and 48 h (Figure 2).

### Cell toxicity assays

**Figure 3:**
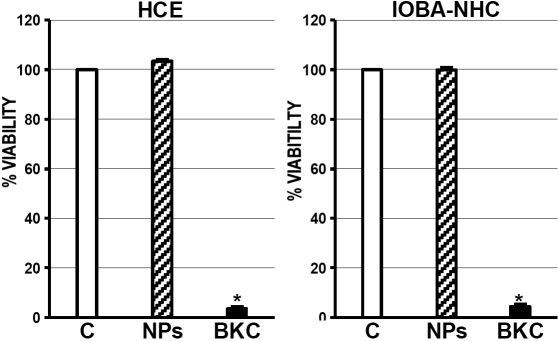
Cell viability 48 h after HA-CSO NPs treatment. Results are expressed as % of control (C). Benzalkonium cloride (BKC) was use as toxicity control. There were no significant differences between NP-treated and non-treated corneal and conjunctival cells (n=4).

Cell viability was measured in both cell lines 48 h after removing the HA-CSO NPs. As expected, BKC-exposed cells showed a very low viability percentage (Figure 3). However, there were no significant differences in cell viability between control corneal and conjunctival cell lines and those exposed to 20 µg/ml of pSEAP-loaded HA-CSO NPs.

### Trafficking of HA-CSO NPs

To rule out phagocytosis as a potential mechanism for HA-CSO NP uptake, the phagocytic capacity of corneal and conjunctival cell lines was determined by fluorescent opsonised *E. coli* bioparticles. The fluorescence associated with phagocytosed bioparticles in corneal and conjunctiva cell lines, 11.36±0.58 RFU and 10.45±0.46 RFU respectively, was significantly lower than in the mouse macrophage RAW264.7 cell line, 85.831±10.82 RFU. Control uptake without bioparticles was 12.41±0.78 RFU. Fluorescence microscopy showed abundant *E. coli* bioparticles inside the RAW264.7 cells, whereas their intracellular presence in corneal and conjunctival cell lines were almost nil (data not shown). Therefore, HCE and IOBA-NHC cells have negligible phagocytic capacity.

**Figure 4:**
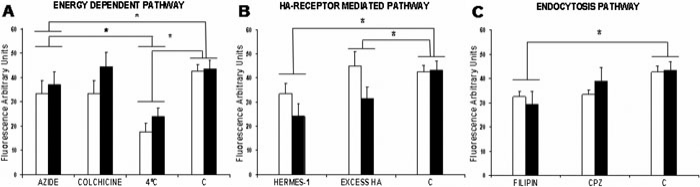
Inhibition of HA-CSO NP uptake. NP uptake was measured by fluorometry. **A**: Both azide and 4 °C significantly decreased NP uptake in HCE (black bars) and IOBA-NHC (white bars) cells. **B**: Blocking the HA-receptors with Hermes-1 or with an excess of HA resulted in a significant reduction of NP uptake. **C**: Filipin, a specific caveolin-dependent endocytosis inhibitor, significantly reduced HA-CSO NPs uptake in both cell lines while CPZ, a specific clathrin-dependent endocytosis inhibitor, did not (*p-value<0.05; n=4).

Endocytosis can be strongly inhibited with depletion of cellular energy resources by lowered temperatures and with metabolic inhibitors. The corneal and conjunctival cell lines were incubated with HA-CSO NPs at 4 °C or with sodium azide or colchicine at 37 °C. Uptake was significantly reduced by incubation at 4 °C and by sodium azide (Figure 4A), both of which inhibit active transport processes. Inhibition of NP uptake by low temperature was significantly greater than that by azide (p<0.05, Figure 4A). Colchicine did not reduce HA-CSO NP uptake.

To determine if the main HA receptors, CD44 and RHAMM, were responsible for the cellular uptake of HA-CSO NPs, we blocked them with an excess of HA and with the anti-CD44 monoclonal antibody Hermes-1. HA-CSO NP uptake was significantly reduced by Hermes-1 and excess HA (Figure 4B). While the reduction in uptake by Hermes-1 appeared to be more effective that excess HA, there were no significant differences between them.

To determine the kind of endocytic pathway implicated in the HA-CSO NP uptake, cells were incubated with chlorpromazine (CPZ) to inhibit clathrin-dependent endocytosis and filipin to inhibit caveolin-dependent endocytosis. CPZ did not significantly affect HA-CSO NP uptake by either corneal or conjunctival cells (Figure 4C). However, the uptake of HA-CSO NPs in both cell lines was significantly inhibited by filipin. Thus HA-CSO NP uptake is mediated mainly by caveolin-dependent endocytosis.

### Caveolin-1 and clathrin co-localization with HA-CSO NPs

**Figure 5:**
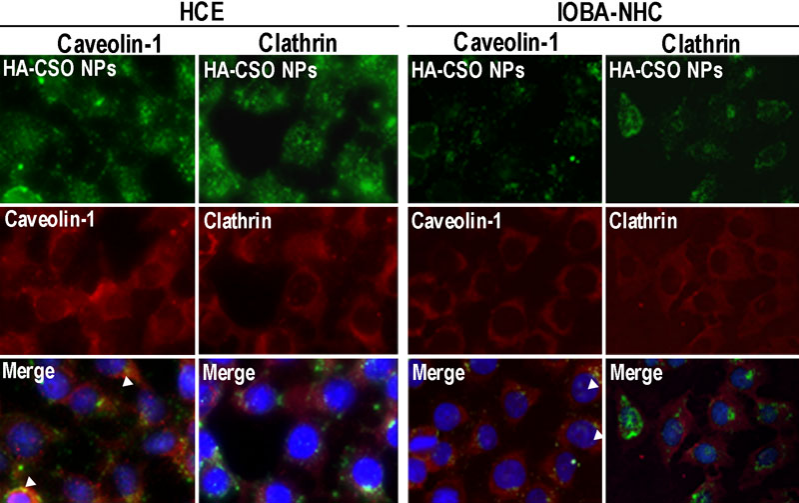
Caveolin-1 and clathrin immunofluorescence in HCE and IOBA-NHC cells after HA-CSO NP incubation. Caveolin expression was higher than clathrin expression. Merged images showed co-localization of HA-CSO NP with caveolin (staining at arrowheads). Representative images of four different experiments are shown.

To further confirm the involvement of caveolin-mediated processes in the uptake of HA-CSO NPs, we determined if the staining pattern of caveolin-1 was associated with the NP fluorescence. Co-localization of NPs and caveolin-1 was present in both cell lines at the plasma membrane and in the cytoplasm, probably due to internalized caveolin vesicles (Figure 5). In contrast, while clathrin was present in the corneal and conjunctival cell lines, there was no remarkable co-localization between it and the NPs. These results are consistent with the inhibition of uptake by filipin and the lack of inhibition by CPZ (Figure 4C).

### Degradative pathways

**Figure 6:**
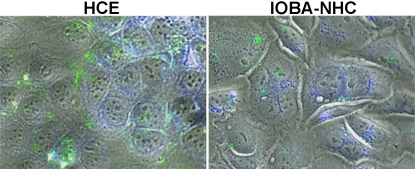
Lysosensor staining in HCE and IOBA-NHC cells. At 24 h after incubation, both HA-CSO NPs (green) and Lysosensor-stained lysosomes (blue) were present in both cell lines. However there was no evidence of NP colocalization with lysosomes. Representative images of four different experiments are shown.

To determine if the loss of fluorescence 6 h after uptake (Figure 2) was due to lyosomal degradation, cells were incubated with NPs and then treated with LysoSensor, a marker of lysosomes. NPs were not co-localized with LysoSensor in the lysosomal compartment after 24 (Figure 6) or 48 h. The presence of green fluorescent NPs in the cytoplasm suggested that they were not subject to lysosomal degradation. Altogether, these data implicated an active NP degradation by a lysosome-independent process at 24 and 48 h.

### HA-CSO NP transfection efficiency

**Figure 7:**
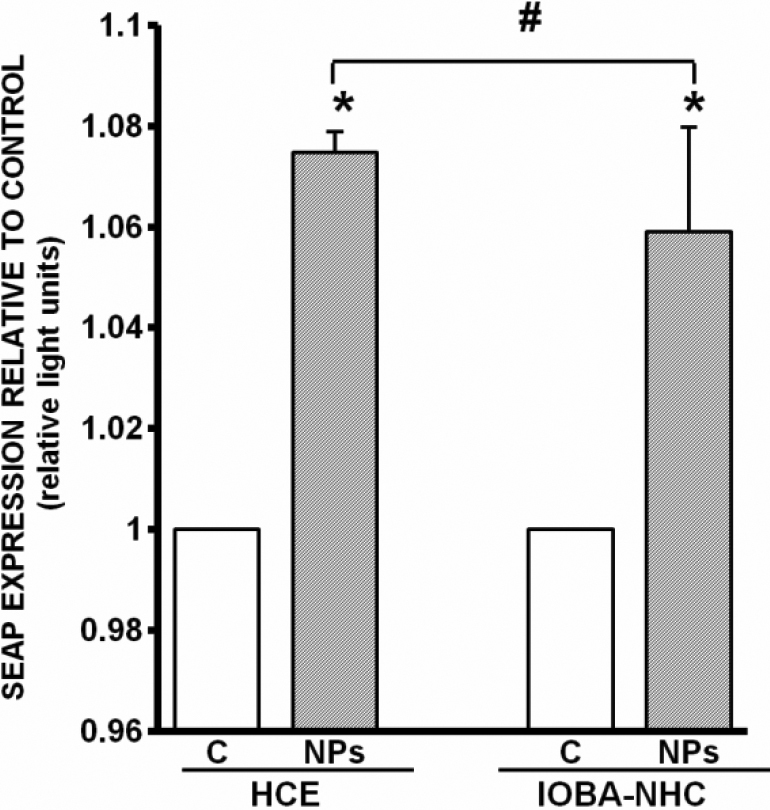
Evaluation of NP transfection efficiency. Transfection levels were significantly higher in pSEAP-loaded HA-CSO NP-transfected cells (NPs) than in control cells (C; *p<0.05). HCE transfection efficiency was significantly higher than IOBA-NHC efficiency (#p<0.05; n=4).

The capacity of the HA-CSO NPs to transfect cells was evaluated 48 h after NP incubation. In this experiment we measured the concentration of SEAP in cells exposed to HA-CSO NPs. pSEAP in solution alone showed negligible transfection levels (data not shown). However, the transfection levels in cells incubated with HA-CSO NPs containing pSEAP were significantly increased compared with controls (Figure 7). Transfection efficiency of the corneal cell line was significantly higher than that of conjunctival cell line.

## Discussion

NPs are alternative drug delivery systems that have the potential for effective delivery of genetic material to the eye. There are several studies in which NPs have been used for ocular gene delivery [27,28], but most of them have problems associated with toxicity, an invasive administration route, or low ability to reach the target cells. However, we have reported that HA-CSO NPs have a suitable composition, size, and Z-potential to ensure interaction with the ocular epithelia [2], and that they are well tolerated by the ocular surface [11].

In this work we studied the different pathways that may be responsible for HA-CSO NP uptake and degradation by human epithelial cell lines derived from the ocular surface. We also evaluated the transfection capability of pSEAP-loaded HA-CSO NPs. For the two cell lines, HCE and IOBA-NHC, HACSO NPs were internalized mainly by an active transport mechanism mediated by HA receptors through a caveolin-dependent endocytic pathway. Fluorescence microscopy and intracellular NP-associated fluorescence quantification showed a remarkable intracellular localization of HA-CSO NPs in both cell lines. These results are consistent with our previous work with CS NPs that showed a similar intracellular distribution [5].

Importantly, the plasmid-associated fluorescence was localized in the nucleus or in the perinuclear region after 24 and 48 h. This localization is crucial to achieve good transfection efficiency. On internalization, HA is included in the non-lysosomal vesicle compartment and is rapidly accumulated in the perinuclear region and in cell nuclei [29,30]. Therefore, the HA component of HA-CSO NPs may use the same type of mechanism to lead the plasmid to the nuclear compartment.

Although the differences were not significant, the ability of HA-CSO NPs to enter the cornea-derived HCE cells was higher than for conjunctiva-derived IOBA-NHC cells. This difference could be associated with the expression of mucins on the surface of the IOBA-NHC cells. This mucoid component might hamper the interaction with the cellular membrane and therefore the internalization of the NPs in IOBA-NHC cells, as we have shown in previous studies [31]. The minimal difference in uptake for the two cell lines may be due to the presence of HA in the NPs. This could improve the interaction of the NPs with the cells and enhance internalization.

The intracellular presence of HA-CSO NPs decreased as the time of incubation increased. This suggests an active process of intracellular degradation, perhaps mediated by non-lysosomal enzymes known to catabolize HA and CS, such as hyaluronidases [32], chitinases [33], and lysozymes [34]. These enzymes are present in the ocular surface tissues [35-37]; however, their expression in the cell lines used for this study needs further clarification.

The viability of treated corneal and conjuctival cell lines was totally preserved. This is in agreement with previous in vitro [2] and in vivo [11] works for these nanosystems. It indicates an adequate level of safety for the application of HA-CSO NPs in the ocular surface. Other evidences of the safe application of NPs to the ocular surface have been reported [38].

For any drug, understanding the initial mode of internalization is the first step in achieving optimized drug delivery. HA-CSO NPs uptake is energy-dependent as shown by its inhibition with low temperature and sodium azide. This is consistent with previous works that indicate that cargo uptake is usually energy-dependent [13]. Internalization of substances typically occurs by macropinocytosis, phagocytosis, clathrin-dependent endocytosis, caveolin-mediated endocytosis, or clathrin- and caveolin-independent pathways [15]. We did not study the macropinocytosis pathway because it involves internalization of relatively large particles (1–5 μm diameter), much larger than the HA-CSO NPs, which averaged 104.6 nm. Phagocytosis is usually associated with specialized cells [12], although some epithelial cells are also capable of it [39]. However, we found that the capacity for phagocytosis of the ocular surface epithelial cells in vitro was negligible.

According to our previous results, the presence of HA in the NPs would ease access to corneal and conjunctival epithelial cells that express the hyaluronan receptor CD44. Accordingly, we found that HA-CSO NP uptake was blocked by the monoclonal antibody Hermes-1 for the CD44 receptor as well as by an excess of HA, suggesting that these NPs enter the cell by a receptor-mediated process. CD44 is the primary cell surface receptor for HA in the ocular surface epithelia [17], and it binds HA with five times greater affinity than RHAMM [40]. Excess HA also decreased NP uptake, but the inhibition was somewhat less than that by Hermes-1 antibody, possibly due to more effective blocking by the monoclonal antibodies. Recently, we found that CD44 expression is higher in human corneal than in conjunctival tissues (unpublished). This is consistent with the higher uptake of HA-CSO NP by corneal than by conjunctival cell line. Therefore, there may be a correlation between CD44 expression and HA-CSO NP uptake. RHAMM also has the capability to bind HA, although cell surface CD44 function is dominant [41]. Even though we cannot rule out the participation of RHAMM in the HA-CSO NP uptake, it seems to be much less important than CD44.

Endocytosis is a basic cellular process that is used by all cell types to internalize a variety of molecules by means of two different specialized proteins: clathrin and caveolin [15]. CPZ, a specific inhibitor of clathrin-dependent endocytosis, did not significantly affect HA-CSO NP uptake. In contrast, filipin, a specific inhibitor of caveolin-dependent endocytosis, significantly reduced NP uptake. In addition, attempts to co-localize antibody-labeled clathrin with the NPs were not successful, while antibody-labeled caveolin-1 did co-localize with HA-CSO NPs. Caveolin-dependent endocytosis occurs quickly, in less than 20 min [13]. Our results are in agreement with that, as we observed an abundance of NPs inside the cells after 1 h incubation. These findings suggest that HA-CSO NPs are mainly internalized by the caveolin-mediated endocytosis pathway.

The degradation pathway of NPs following internalization is another important aspect of intracellular NP dynamics. Degradation by lysosomes is a common terminal destination for the endocytic pathway [19]. However, lysosomes and HA-CSO NPs were not co-localized in the cytoplasm after 24 and 48 h, suggesting that vesicles containing the NPs probably were not directed there following uptake. However, it is possible that there was a degree of elimination by lysosomes before 6 h. The CD44 receptor and HA are associated with caveolae, and the caveolar internalization pathway bypasses lysosomes [42]. Thus it is not surprising that we found no evidence of NP degradation by lysosomes.

**Figure 8:**
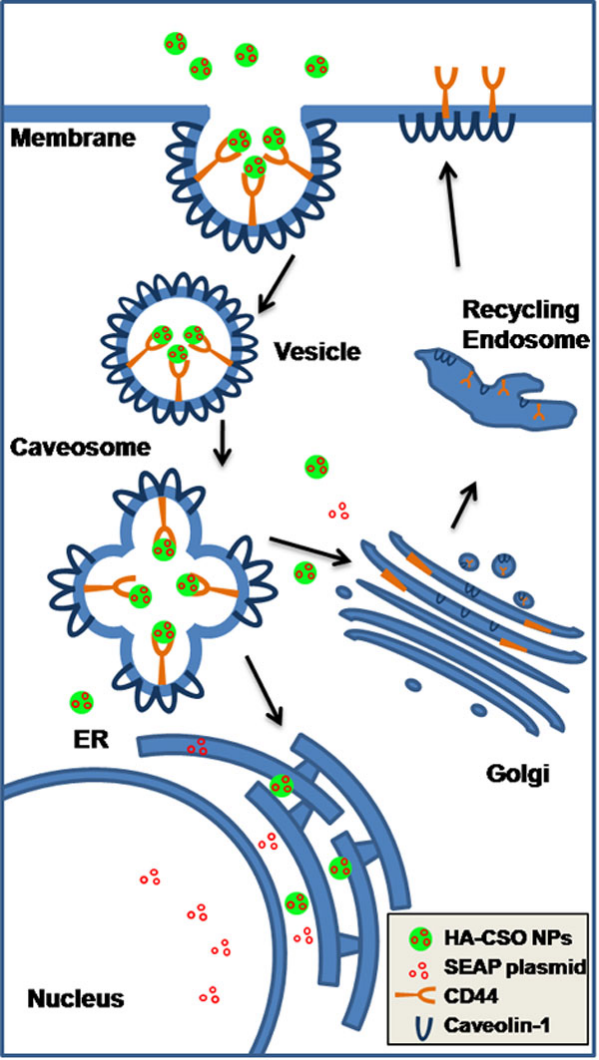
Proposed HA-CSO NP internalization pathway. HA-CSO NPs interact with CD44 receptors in the plasma membrane, triggering the internalization of the caveola vesicles that then fuse with the caveosome. From the caveosome, NPs are sorted to the endoplasmic reticulum (ER) and then to the nucleus. CD44 receptors and caveolin are then recycled and carried back to the membrane through the Golgi network.

Based on these data, we propose that the main HA-CSO NP uptake pathway begins with the interaction of the HA component of the HA-CSO NPs and CD44 followed by caveolae internalization. Caveosomes are large subcellular organelles characterized by a neutral pH and caveolin, which is responsible for sorting the vesicle contents [42]. Internalized caveolae loaded with HA-CSO NPs may fuse with caveosomes that then deliver the NPs into other subcellular compartments, typically to the Golgi network or to the ER and subsequently to the nucleus (Figure 8). More studies are needed to clarify the complete pathway.

The final goal of a gene delivery system is to produce a significant level of gene transfection. HA-CSO NPs loaded with SEAP pDNA yielded significant transfection of the corneal and conjunctival cell lines. Transfection efficiency of the HCE cells was significantly higher than that of the IOBA-NHC cells. Taking into account that NP uptake was also higher in HCE cells, we believe that the internalization process is the limiting step for the efficiency of these delivery systems as transfection vehicles. At least for the cell lines studied, HA-CSO NPs are a suitable gene delivery system.

To conclude, we propose that HA-CSO NPs are internalized by corneal and conjunctival cell lines by an active transport mechanism mediated mainly by the CD44 HA receptor through a caveolin-dependent endocytic pathway. Using this pathway, plasmid-loaded HA-CSO NPs achieved significant transfection efficiency. These NPs did not compromise cell viability and most of them were metabolized by the ocular surface epithelial cell lines in 48 h. These facts further support the potential use of HA-CSO NPs to deliver genetic material to the ocular surface.
